# Significance of Dynamic Axial Stretching on Estimating Biomechanical Behavior and Properties of the Human Ascending Aorta

**DOI:** 10.1007/s10439-024-03537-6

**Published:** 2024-06-05

**Authors:** Shaiv Parikh, Alessandro Giudici, Wouter Huberts, Tammo Delhaas, Elham Bidar, Bart Spronck, Koen Reesink

**Affiliations:** 1 Department of Biomedical Engineering, CARIM School for Cardiovascular Diseases, Maastricht University, Maastricht, The Netherlands; 2 GROW School for Oncology and Reproduction, Maastricht University, Maastricht, The Netherlands; 3 Department of Biomedical Engineering, Cardiovascular Biomechanics, Eindhoven University of Technology, Eindhoven, The Netherlands; 4 Department of Cardiothoracic Surgery, Heart & Vascular Centre, Maastricht University Medical Centre, Maastricht, The Netherlands; 5 Faculty of Medicine, Health and Human Sciences, Macquarie Medical School, Macquarie University, Sydney, Australia

**Keywords:** Ascending thoracic aorta, Biaxial stretching, Biomechanical response, Parameter estimation

## Abstract

Contrary to most vessels, the ascending thoracic aorta (ATA) not only distends but also elongates in the axial direction. The purpose of this study is to investigate the biomechanical behavior of the ascending thoracic aorta (ATA) in response to dynamic axial stretching during the cardiac cycle. In addition, the implications of neglecting this dynamic axial stretching when estimating the constitutive model parameters of the ATA are investigated. The investigations were performed through in silico simulations by assuming a Gasser–Ogden–Holzapfel (GOH) constitutive model representative of ATA tissue material. The GOH model parameters were obtained from biaxial tests performed on four human ATA tissues in a previous study. Pressure–diameter curves were simulated as synthetic data to assess the effect of neglecting dynamic axial stretching on estimating constitutive model parameters. Our findings reveal a significant increase in axial stress (~ 16%) and stored strain energy (~ 18%) in the vessel when dynamic axial stretching is considered, as opposed to assuming a fixed axial stretch. All but one artery showed increased volume compliance while considering a dynamic axial stretching condition. Furthermore, we observe a notable difference in the estimated constitutive model parameters when dynamic axial stretching of the ATA is neglected, compared to the ground truth model parameters. These results underscore the critical importance of accounting for axial deformations when conducting in vivo biomechanical characterization of the ascending thoracic aorta.

**Supplementary Information:**

The online version contains supplementary material available at 10.1007/s10439-024-03537-6.

## Introduction

Various studies have demonstrated the significance of arterial stiffening as a predictor of cardiovascular diseases in humans [1–3]. Carotid-to-femoral pulse wave velocity is considered the current gold standard for measuring arterial stiffness in vivo [1]. However, it quantifies the average arterial stiffness over the entire arterial pathway between the carotid and femoral artery. Other clinical measures, such as the pressure–strain modulus or Peterson modulus (the inverse of distensibility), allow for determining local stiffness of a single artery [1, 4]. However, both types of metrics provide an evaluation of arterial stiffness at a global (or structural) level [1, 4], which reflects a combination of both micro (e.g., wall tissue composition and microstructural arrangement of constituents) and macro-structural vessel properties (e.g., arterial diameter and thickness). Because vascular aging and disease may alter both classes of vessel properties, structural stiffness metrics only capture their overall effect on arterial function (macro-structural level) but are ill suited to provide an in-depth mechanistic understanding of the associated degenerative processes (microstructural level) [4–7].

Previous studies have addressed this limitation by employing microstructurally motivated constitutive models to capture the in vivo material properties of arteries [4]. Microstructurally motivated constitutive models [8–10] encompass the elastic properties of load-bearing constituents within the arterial wall, which include elastin, collagen fibers, and smooth muscle cells. These constituents collectively contribute to the artery's overall elastic behavior. Previous studies have attempted to estimate material parameters of microstructurally motivated models from human in vivo data [4, 11, 12]. The constitutive model parameters in those studies were estimated by assuming the vessel to be a homogeneous cylinder and by minimizing the error between the computed and experimental data. Typically, the experimental data consist of pressure–diameter relationships derived from medical images and tonometer- or catheter-based measurements. However, because of the anisotropy of the microstructurally motivated constitutive models, often an implicit assumption on the vessel behavior in the axial direction (e.g., constancy of reduced axial force in the physiological range) is made and involved in the parameter estimation.

A pressure–diameter relationship adequately describes the vessel kinematics of most large arteries in our body (e.g., the carotid arteries and abdominal aorta) which are maintained at a fixed axial stretch throughout the cardiac cycle. However, it is acknowledged that the ascending aorta also undergoes dynamic axial stretching due to its physical connection to the pumping heart [13–17]. Because this axial stretching is comparable in magnitude to that the ascending aorta experiences in the circumferential direction [17], it is reasonable to assume that it holds a significant importance in determining the physiologically relevant constitutive model parameters of the ascending aorta. These parameters, in turn, play a pivotal role in influencing the aorta's physiological function such as the Windkessel effect [18, 19]. Until now, this ascending aortic axial deformation has been neglected while estimating constitutive material parameters [12], which could lead to their inaccurate estimation [20].

The aim of this study is twofold. *First*, we aim to quantitatively evaluate the effects of dynamically varying axial stretches (DVAS) on the biomechanical response of the ascending aorta. To this end, we use the constitutive models of human ascending aortas provided in Smoljkić et al. [12] and the intra-operative strain measurements previously published by our group [17]. We then simulate the response of the ascending aorta to a physiological loading scenario and compare this to that obtained when assuming the vessel to be at a fixed axial stretch (FAS). *Second*, using the generated synthetic data in the form of pressure–diameter curves, we aim to establish the impact of neglecting this dynamic axial stretching on the estimation of the model parameters of the ascending aorta. With this study, we emphasize the relevance of including imaging-based patient-specific dynamic axial stretches in in vivo parameter estimation studies.

## Methods

### Geometrical Data

The human ascending aorta was modeled as an incompressible, idealized, single-layered, thin-walled cylinder. Data of four cylindrical geometries were obtained from Smoljkić et al. [12], who investigated the biaxial mechanical behavior of the human ascending aortic aneurysm using tissue from patients undergoing aortic repair surgeries. In their work, three to six samples of each patient’s aorta were subjected to mechanical characterization via planar biaxial testing. For each geometry, the unloaded wall thickness ($H$) was obtained by averaging sample-wise values from Table 5 in [12]. The unloaded internal radius ($R$) was derived in such a way that upon pressurization to the patient-specific diastolic pressure (Table 1 in [12], see Sect. "Boundary Conditions"), the simulated internal radius matched patient-specific in vivo values reported in Table 1 from [12]. Additionally, the unloaded axial length of all vessels was assumed to be 40 mm for all arteries.

### Microstructure-Based Constitutive Model and Parameters

A Gasser–Ogden–Holzapfel (GOH) constitutive model (Eq. 1) was used in [12] to represent the incompressible material behavior of the tissue. The strain energy density function for the GOH constitutive model ($\psi$) is given by an additive decomposition of an isotropic contribution of matrix material ($\psi_{\text{mat}}$) and the anisotropic contribution from two collagen fiber families (${\psi }_{\text{col}}$):

1 $$\begin{array}{c}\psi = {\psi }_{\text{mat}}+ {\psi }_{\text{col}}\end{array},$$

2 $$\begin{array}{c}{\psi }_{\text{mat}}=\frac{\mu }{2}\left({I}_{1}-3\right), {\text{and}}\end{array}$$

3 $$\begin{array}{c}{\psi }_{\text{col}}=\frac{{k}_{1}}{{2k}_{2}}\sum_{i=\text{4,6}}\left\{\text{exp}\left\{{k}_{2}{{[(\kappa I}_{1}+(1-3\kappa ) {I}_{i})-1]}^{2}\right\}-1\right\} ,\end{array}$$

($\mu$ is a stiffness-like parameter of the isotropic matrix material, ${k}_{1}$ is a stiffness-like parameter associated with collagen fibers, ${k}_{2}$ is the dimensionless parameter related to the nonlinear stiffening of collagen fibers, and $\kappa$ is the dispersion of collagen fibers),

where

4 $$\begin{array}{c}{I}_{1}=\text{tr}\left(\mathbf{C}\right),~~{I}_{4}=\mathbf{C}:\mathbf{m} \otimes \mathbf{m},~~{\text{and}}~~{I}_{6}=\mathbf{C}:\mathbf{n} \otimes \mathbf{n} ,\end{array}$$

where $\text{tr}()$, :, and $\otimes$, represent the trace of a second-order tensor, double dot product of second-order tensors, and dyadic product of two vectors, respectively, 

with

5 $$\begin{array}{c}\mathbf{F}= \left[\begin{array}{ccc}{\lambda}_{r}& 0& 0\\ 0& {\lambda}_{\theta }& 0\\ 0& 0& {\lambda}_{z}\end{array}\right] ,~~\mathbf{C}= {{\mathbf{F}}}^{\text{T}}\mathbf{F} ,\end{array}$$

6 $$\begin{array}{c}\mathbf{m}={\left[\begin{array}{ccc}0& \text{cos}\alpha & \text{sin}\alpha \end{array}\right]}^\text{T},~~{{\text{and}}}\,~~\mathbf{n}={\left[\begin{array}{ccc}0& \text{cos}\alpha & -\text{sin}\alpha \end{array}\right]}^\text{T} .\end{array}$$

where ${\mathbf{F}}$ is the deformation gradient tensor of a homogenously inflated and extended thin-walled cylinder with stretches in the radial, circumferential, and axial directions as ${\lambda}_{r}$, ${\lambda}_{\theta },$ and ${\lambda}_{z}$, respectively, $\alpha$ is the orientation of collagen fibers in the circumferential-axial plane with respect to the circumferential direction, and []^T^ indicates the transpose of a tensor or vector.

The material parameters of the four human ascending aortas are obtained from Table 1 in [12]. In their work, three to six samples of each artery were subjected to ex vivo biaxial mechanical characterization, and one set of GOH model parameters was fit to each individual response, thus, yielding three to six sets of parameter values per artery. To obtain a representative set of model parameters for each artery, we used each of its sets of model parameters to simulate its response to biaxial tensile testing with circumferential:axial stretching ratios 0.5:1, 0.75:1, 1:1, 1:0.75, and 1:0.5 [12]. We then ensemble averaged the obtained responses to yield a representative average response for each of the circumferential:axial stretching ratio. Finally, we determined a new set of “averaged” material parameters by re-fitting the material model to the obtained averaged response. The parameter estimation was performed using the MATLAB function *lsqnonlin* (R2023a, MathWorks, Natick, MA, US) and minimizing the difference in circumferential and axial Cauchy stresses between the averaged response and that obtained with the new set of model parameters.

### Boundary Conditions

For each vessel, two conditions were simulated, namely fixed axial stretch (FAS) and dynamically varying axial stretch (DVAS). Simulating both conditions involved initially setting a fixed axial stretch of 1.2 and identifying the circumferential stretch that resulted in a luminal pressure equal to the patient-specific diastolic pressure (Table 1). Note that because the patient-specific in vivo axial stretches with respect to the zero-pressure configuration were not known [21], a value of 1.2 was assumed based on the data presented in a previous study on humans [22]. The pressure was assumed to be acting normal only to the inner surface of the artery and was determined from Laplace’s law:

7 $$\begin{array}{c}{P}_{\text{model}}=\frac{{\sigma }_{\theta \theta } \cdot h}{r},\end{array}$$

where $h={\lambda}_{r}H$ is the loaded wall thickness, $r={\lambda}_{\theta }R$ is the loaded inner radius, and ${\sigma }_{\theta \theta }$ is the circumferential Cauchy stress. Given Eqs. 1–6, ${\sigma }_{\theta \theta }$ follows from

8 $$\begin{array}{c}\varvec\sigma =2 \mathbf{F}\frac{\partial \psi }{\partial \mathbf{C}}{{\mathbf{F}}}^\text{T}-p\mathbf{I},\end{array}$$

where ${\varvec{\sigma}}$ is the Cauchy stress tensor and $p$ is a Langrange multiplier which enforces incompressibility and can be determined from the plane stress condition, i.e., radial Cauchy stress is zero (${\sigma }_{rr}=0)$, and ${\mathbf{I}}$ is the second-order identity tensor.

**Table 1:** Patient-specific arterial in vivo pressures obtained from [12]

Patient	Diastolic pressure (mmHg)	Systolic pressure (mmHg)
F68	99	149
M58	65	135
M60	84	135
M55	73	144

In the FAS condition, the axial stretch of 1.2 was maintained constant throughout the cardiac cycle, and the vessel was further pressurized until reaching the patient-specific systolic pressure (Table 1). In contrast, in the DVAS condition, we applied an extra 4% engineering axial strain to the vessel and simultaneously raised the pressure to mimic the physiological response at systole [17]. In this scenario, we assumed the axial stretch to increase linearly with pressure so that the total axial stretch was 1.2 and 1.2·1.04 at end diastole and peak systole, respectively.

### Calculation of Biomechanical Parameters of Interest

To compare the biomechanical behavior of the ascending aorta when subjected to the DVAS vs. FAS-loading conditions, material and structural biomechanical variables of interest were determined for both loading conditions. All calculations were performed analytically in MATLAB (R2023a, MathWorks, Natick, MA, US).

#### Cauchy Stress and Material Stiffness

For each vessel, the circumferential and axial Cauchy stresses were calculated at systolic pressure using Eq. (8). Due to thin-walled cylinder consideration, the Cauchy stress in the radial direction was neglected. The circumferential and axial material stiffness values at systolic pressure were calculated based on the small-on-large theory [23]:

9 $$\begin{array}{c}{\mathbb{C}}_{iiii}=2 \left({\sigma }_{\text{sys},ii}+p\right)+4{{\lambda}^{4}_{\text{sys},i}}{\left.\frac{{\partial }^{2}\left(\psi \right)}{\partial {\lambda}_{i}^{2}\partial {\lambda}_{i}^{2}}\right|}_{\text{sys}},\end{array}$$

where ‘$\text{sys}$’ represents quantities calculated at systolic pressure, and $i= \theta , z$, i.e., representing the circumferential and axial directions, respectively. Here, the repeated indices (‘$i$’) are not to be confused with Einstein summation.

#### Stored Energy and Volume Compliance

The stored elastic energy and volume compliance over a single cardiac cycle were calculated as differences between systolic and diastolic values. The stored energy is expressed as follows:

10 $$\begin{array}{c}\Delta \psi = {\psi }_{\text{sys}}- {\psi }_{\text{dias}} , \end{array}$$

where ‘$\text{dias}$’ indicates diastole. The volume compliance was calculated using

11 $$\begin{array}{c}{C}_{\text{v}}= \frac{\Delta V}{\Delta P} , \end{array}$$

where $\Delta V$ is the difference in intra-luminal volume between systole and diastole, and $\Delta P$ is the pulse pressure. Note that $\Delta V$ accounts for both changes in diameter and axial length of the artery within a cardiac cycle.

#### Statistical Analysis

Paired sample Student’s *t*-tests were performed to evaluate differences in biomechanical parameters of interest between the FAS and DVAS loading conditions. *p* < 0.05 was considered statistically significant.

### Parameter Estimation from Synthetic Experimental Data

To quantify the effect of neglecting the dynamic axial stretching of the ascending aorta on the estimation of constitutive model parameters, we used the known model parameters of each vessel (as described in Sect. "Microstructure-Based Constitutive Model and Parameters") to generate pressure–diameter curves for the DVAS condition. The pressure–diameter curve for each vessel was assumed to be the ground truth of the physiological behavior of the vessel in vivo and is hereafter referred to as synthetically acquired experimental data. These data were then used to test the accuracy of the parameter estimation when considering the arteries undergoing FAS and DVAS types of deformation, respectively.

In total, there are seven parameters that are required to be estimated while fitting the constitutive model described in Eqs. 2 and 3 to in vivo data. Those parameters are $\mu$, ${k}_{1}$, ${k}_{2}$, $\alpha$, and $\kappa$, unloaded mid-membrane diameter ${D}_{0}$, and the fixed axial stretch at diastolic pressure ${\lambda}_{z,\text{dias}}$ (which, as described in Sect. "Boundary Conditions", was considered as 1.2), assuming that in vivo thickness can be measured (e.g., as intima-media thickness). Note that when fitting the model parameters to the synthetic data under the DVAS assumption, the amplitude of the dynamic axial stretching (i.e., a 4% engineering strain) was assumed to be known. This is because such axial deformation can be measured through in vivo imaging (e.g., CT scans) by using anatomical markers on the ascending aorta (e.g., aortic root and brachiocephalic bifurcation). The parameter estimation was performed using the MATLAB function *lsqnonlin* and minimizing the cost function:

12 $$\begin{array}{c}\Pi = \sum_{i=1}^{n}{\left[\begin{array}{c}{\left(\frac{{P}_{\text{model}}-{P}_{\text{synthetic exp}.}}{{\overline{P} }_{\text{synthetic exp}.}}\right)}^{2}\\ \end{array}\right]}_{i}+\frac{n}{10}\cdot {\left(\frac{{\bar{\sigma }}_{\text{zz},\text{ model}}-{\sigma }_{zz,\text{target}}}{{\sigma }_{zz,\text{target}}}\right)}^{2},\end{array}$$

where ${P}_{\text{synthetic exp}.}$ is the synthetic data pressure, ${\overline{P}}_{\text{synthetic exp}.}$ is the mean synthetic data pressure, ${P}_{\text{model}}$ and ${\bar{\sigma }}_{\text{zz},\text{ model}}$ are the pressure and mean axial stress obtained with the new model parameters, ${\sigma }_{zz,\text{target}}$ is a target axial stress defined as 58% of the mean circumferential Cauchy stress in the synthetic data, and $n$ is the total number of data points on the synthetic pressure–diameter curve. This axial-to-circumferential stress ratio was determined from the synthetically derived experimental data and is in agreement with previously reported experimental observations [24]. Note that because no axial data can be measured in vivo (e.g., reduced axial force or axial stress), the axial part of the cost function $\Pi$ was assigned one tenth of the total weight of the circumferential part (i.e., factor $n/10$) [25, 26]. To assess the sensitivity of the parameters on the fitting process, we progressively fitted seven, six, five, and four parameters on the synthetic data while keeping the rest fixed. The parameters that were held constant during the fitting process are reported in Table 2. Following the parameter estimation, the biomechanical responses of the vessel with the newly estimated parameters were determined. Subsequently, the newly estimated parameters and their associated biomechanical responses were compared against the ground truth. The comparison was performed by determining the error between the ground truth model parameters and biomechanical responses, and newly estimated model parameters and biomechanical responses.

**Table 2:** Fixed parameters and their values while performing parameter estimation with seven, six, five, and four parameters

Parameters to be estimated	Fixed parameters and their values	Estimated parameters
7	–	$\mu$, ${k}_{1}$, ${k}_{2}$, $\alpha$, $\kappa$, ${D}_{0}$, ${\lambda}_{z,\text{dias}}$
6	$\kappa =0.25$	$\mu$, ${k}_{1}$, ${k}_{2}$, $\alpha$, ${D}_{0}$, ${\lambda}_{z,\text{dias}}$
5	$\kappa =0.25, \alpha =30^\circ$	$\mu$, ${k}_{1}$, ${k}_{2}$, ${D}_{0}$, ${\lambda}_{z,\text{dias}}$
4	$\kappa =0.25, \alpha =30^\circ , {k}_{1}=35$ kPa	$\mu$, ${k}_{2}$, ${D}_{0}$, ${\lambda}_{z,\text{dias}}$

$\mu$: shear modulus of the isotropic matrix material, ${k}_{1}$: stiffness-like parameter associated with collagen fibers, ${k}_{2}$: dimensionless parameter related to the nonlinear stiffening of collagen fibers, $\alpha$: orientation of collagen fibers in the circumferential–axial plane, $\kappa$: the dispersion of collagen fibers, ${D}_{0}$: unloaded mid-membrane diameter, ${\lambda}_{z,\text{dias}}$: fixed axial stretch at diastolic pressure

## Results

### Averaged Patient-Specific Geometrical Data and Constitutive Model Parameters

Table 3 depicts values of the derived unloaded internal radius and averaged wall thickness of four patients reported in [12].

**Table 3:** Patient-specific arterial unloaded dimensions obtained from [12]

Patient	Unloaded internal radius (${R}_{\text{i}}$) (mm)	Unloaded wall thickness ($H$) (mm)
F68	17.5	2.6
M58	16.8	2.7
M60	16.1	3.0
M55	15.8	3.2

Table 4 indicates the averaged GOH material parameter values obtained after re-fitting the model on the ensemble averaged stress–stretch curves as described in Sect. "Microstructure-Based Constitutive Model and Parameters".

**Table 4:** Patient-specific averaged arterial material parameters obtained from [12]

Patient	$\mu$ [kPa]	${k}_{1}$ [kPa]	${k}_{2}$ [−]	$\alpha$ [°]	$\kappa$ [−]
F68	38.6	28.9	20.5	27.4	0.28
M58	37.1	56.9	9.9	0.0	0.30
M60	43.2	47.1	12.6	0.0	0.28
M55	41.3	22.3	12.4	20.9	0.15

### Effect of Dynamic Axial Stretching on the Biomechanical Response of the Ascending Aorta

**Fig. 1:**
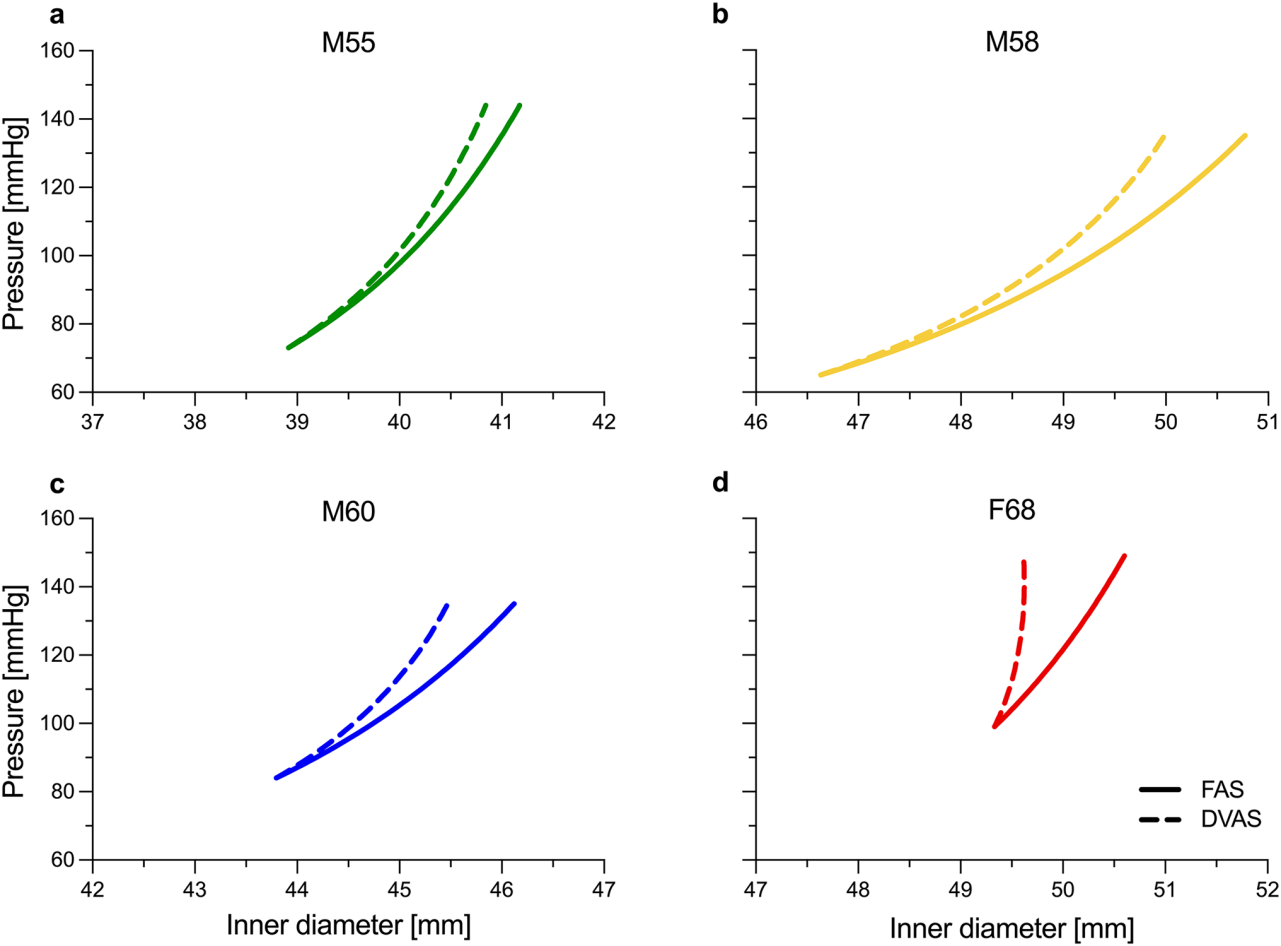
Comparison of pressure–diameter curves between the fixed axial stretch (FAS) and dynamically varying axial stretch (DVAS) loading conditions for the four ascending aortas considered in this study. Note that the *x*-axes of all panels share the same range width (5 mm), thereby facilitating visual comparison of area compliance (i.e., $\Delta A/\Delta P$) between the four aortas

Figure 1 shows the pressure–diameter curves of simulated FAS and DVAS conditions for each artery considered in this study. For the given pressure range (Table 1) and superimposed 4% axial strain, all arteries exhibited monotonically increasing diameters except artery F68 in the DVAS loading condition (Fig. 1d).

**Fig. 2:**
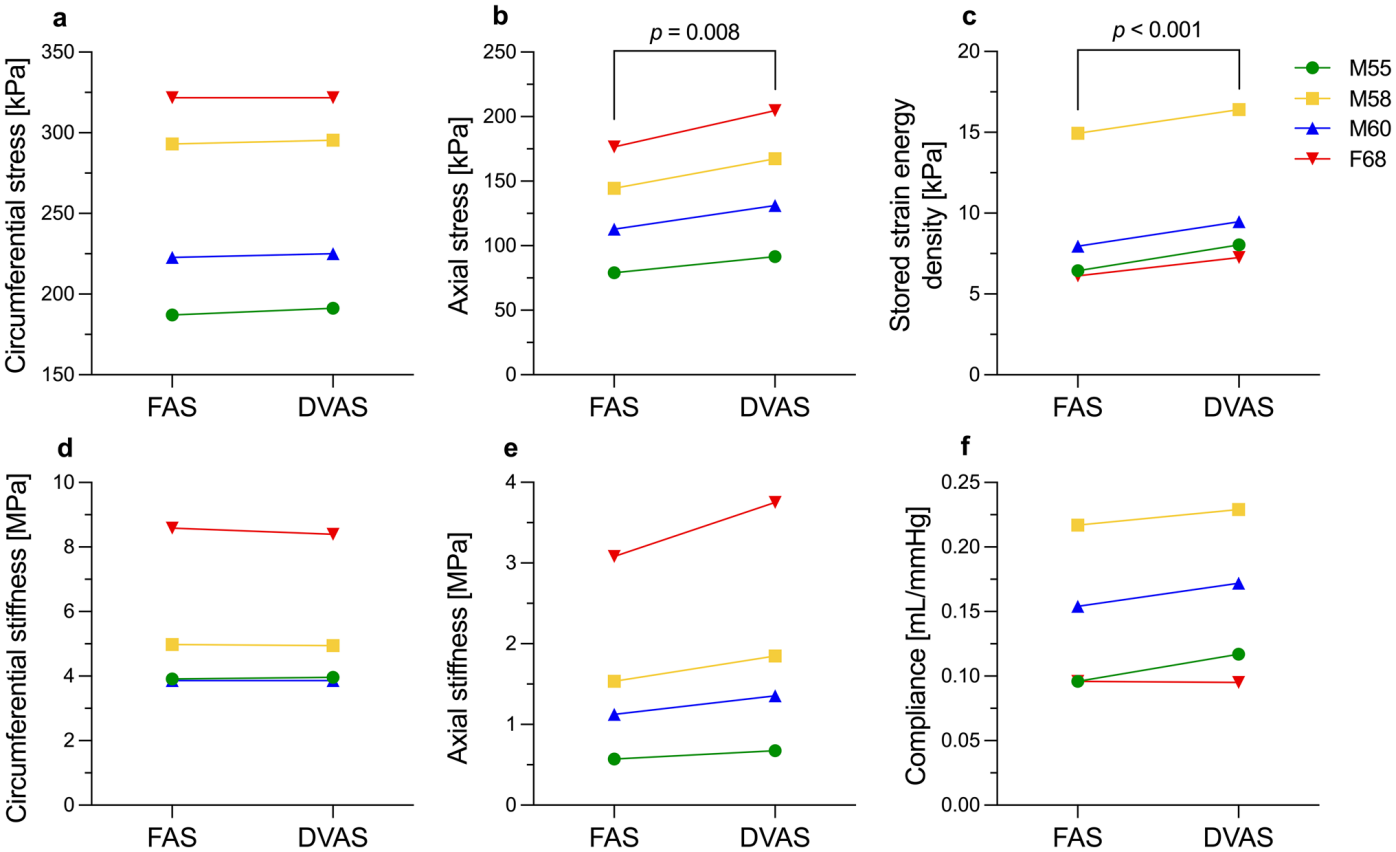
Differences in biomechanical response of the ascending aorta on considering the fixed axial stretch (FAS) and dynamically varying axial stretch (DVAS) conditions

Figure 2 shows the differences in the estimated biomechanical responses between the FAS and DVAS conditions. While the circumferential Cauchy stress (${\sigma }_{\theta \theta }$) and material stiffness (${\mathbb{C}}_{\theta \theta \theta \theta }$) at systole did not vary between the two loading conditions (Fig. 2a, c), the axial Cauchy stress (${\sigma }_{zz}$) and material stiffness (${\mathbb{C}}_{zzzz}$) increased by 15.9 ± 0.2% (*p* = 0.008) and 20.2 ± 1.5% (*p* = 0.073) under the DVAS condition (Fig. 2b, d). In addition, over the considered patient-specific cardiac cycles, the stored strain energy density ($\Delta \psi$) increased by 18.1 ± 6.3% (*p* < 0.001) under the DVAS compared to the FAS condition (Fig. 2e). Similarly, the volume compliance (${C}_{\text{v}}$) increased in all but the stiffest artery (Fig. 2f), which was also the artery showing non-monotonically increasing diameter in the DVAS loading condition (Fig. 1d).

### Impact of Dynamic Axial Stretching on Material Parameter Estimation

**Fig. 3:**
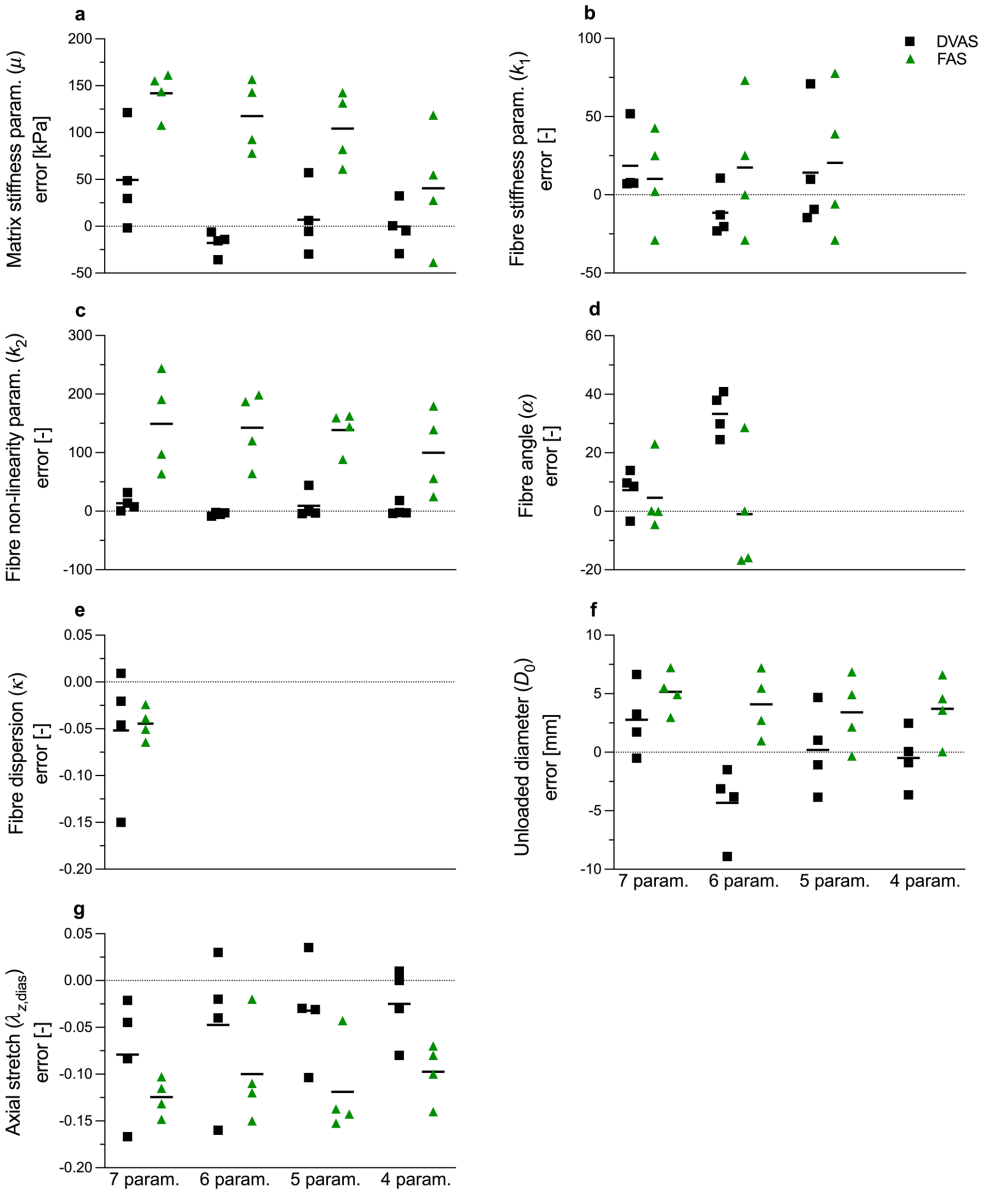
Figure describes progressive reduction in errors in model parameters while lowering the number of parameters. The dynamically varying axial stretch (DVAS) condition exhibited lower errors for all parameters except fiber angle (panel d) as compared to fixed axial stretch (FAS) condition. Errors are calculated by taking the difference of the estimated model parameter from the ground truth model parameter. Horizontal solid black bars represent the mean of errors. The parameters were estimated based on synthetically generated pressure–diameter curves representative of the in vivo condition

Figure 3 illustrates the errors of estimated model parameters under the DVAS and FAS assumptions when estimating seven, six, five, or four parameters. Marked differences were observed while fitting the model with the FAS condition as compared to the DVAS condition. The parameters obtained under the DVAS condition were more representative of the ground truth parameters. Moreover, all the estimated parameters except the axial stretch under the FAS condition were overestimated and showed a much larger between-subject variability compared to the ground truth. Least deviations from the ground truth parameters were observed when only four parameters were estimated. We also observed that the fitting root–mean-square error (RMSE) remains largely unaffected when constraining none to three model parameters (Online Resource, Tables S1–S4).

**Fig. 4:**
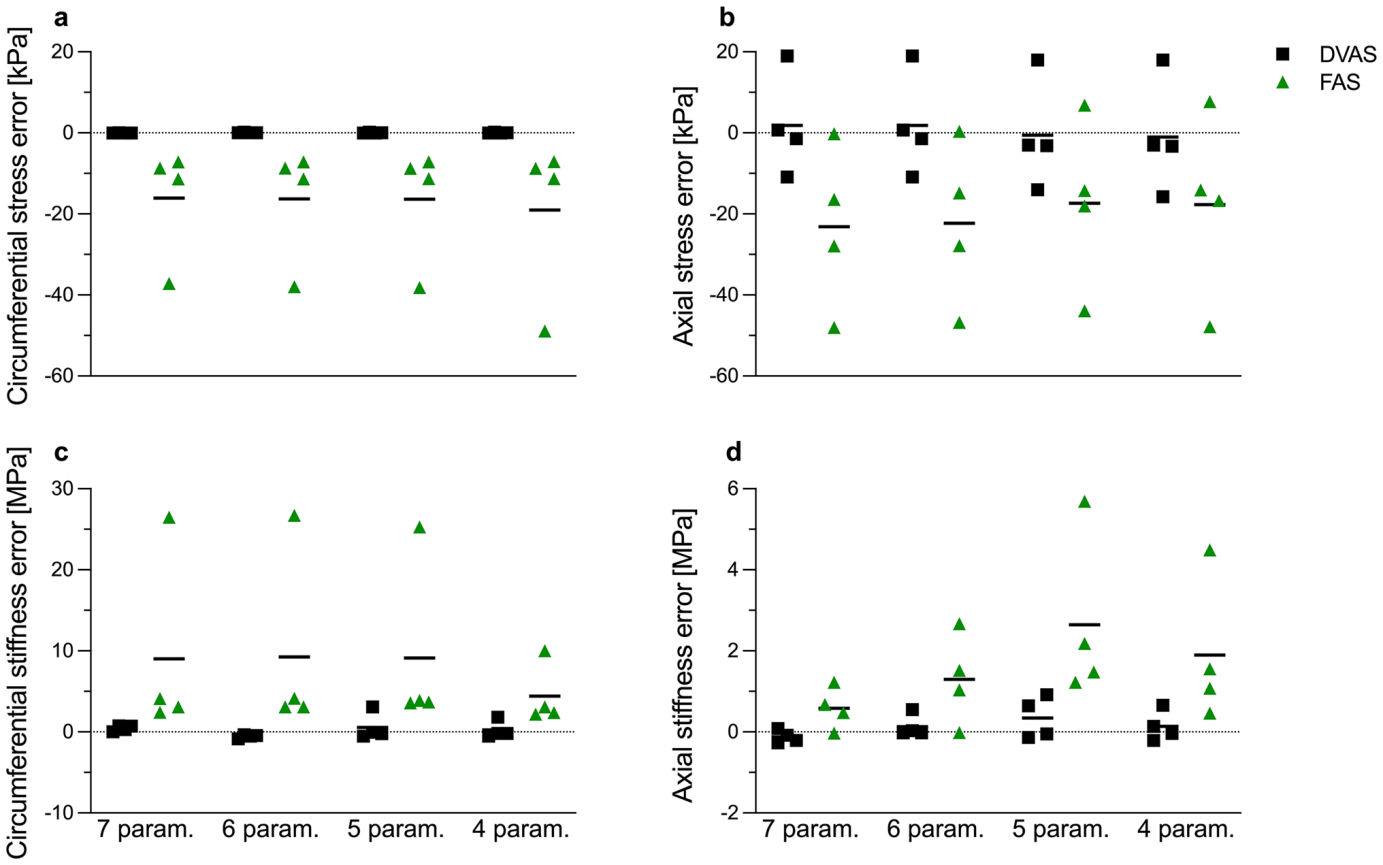
Figure describes errors for biomechanical variables derived from constitutive model parameters estimated by considering fixed axial stretch (FAS) and dynamically varying axial stretch (DVAS) conditions. Biomechanical variables derived under DVAS condition exhibited lower errors as compared to FAS condition irrespective of the number of estimated model parameters. Errors represent deviation from the simulated ground truth biomechanical variables. Mean of errors are represented by black solid horizontal lines

Figure 4 displays the variation of the biomechanical response of the vessels as compared to the ground truth. The variables estimated under the DVAS condition were noticeably comparable to the ground truth variables. Except for the circumferential and axial material stiffness, all the variables were consistently underestimated for the FAS condition.

## Discussion

In this study, we investigated the biomechanical response of the ascending aorta in terms of cyclic axial elongation over a cardiac cycle. We compared this condition to the simplified scenario in which the vessel is assumed to undergo pressurization at a constant axial stretch, which is commonly assumed in most in vivo constitutive modeling studies [12, 27]. Our main findings were that the ascending aorta’s axial material stiffness, compliance, and stored energy predominantly increase when dynamic axial stretching is superimposed to pressurization over the cardiac cycle. Moreover, our study shows that neglecting this dynamic axial deformation leads to significant inaccuracies in the estimation of constitutive model parameters from synthetic data that represent clinically measurable pressure–diameter curves.

Prior research has examined the existence of a cyclic axial elongation of the ascending aorta during a cardiac cycle in both human subjects [13, 15–17] and mice [18]. In humans, axial strain measurements ranged 1–14% across all investigations [13, 15–17]. It has been noted in [13] that axial strain contributes to a proportional decrease in circumferential strain of the ascending aorta. Consequently, it is necessary to account for dynamically changing axial strains to obtain accurate assessments of ascending aortic stiffness [13].

To assess the impact of dynamic axial elongation on the biomechanics of the ascending aorta, we conducted simulations under two loading conditions: FAS, where the aorta was kept at a fixed axial stretch throughout the diastolic-to-systolic pressurization, and DVAS, where an additional 4% axial strain (based on previous intra-operative measurements from our group [17]) was applied between diastole and systole. Despite no discernible differences in circumferential Cauchy stress and material stiffness, the DVAS condition exhibited increased axial Cauchy stress and material stiffness compared to the FAS condition. In addition, the stored energy and volume compliance were higher under the DVAS condition. This highlights a crucial point: if arterial stiffness is assessed solely in the circumferential direction during clinical observation, it may not provide a comprehensive understanding of the ascending aorta's overall structural stiffness, which must also consider stiffness in the axial direction. This fact is exemplified in Figure 1 where evaluating aortic compliance in term of luminal area (i.e., $\Delta A/\Delta P$ with $A$ being the vessel luminal area) yields an apparent decrease in compliance in the DVAS compared to the FAS condition. However, as mentioned earlier, the volume compliance and the stored energy were in fact greater in the DVAS condition compared to the FAS condition. This observation indicates that relying solely on area compliance is insufficient to fully grasp the biomechanical response of the ascending aorta. It is essential to consider the physiological enhancement of the Windkessel effect due to the dynamic axial elongation resulting as a crucial factor [18].

Despite the available evidence that the ascending aorta dynamically elongates during a cardiac cycle, studies that seek to characterize the biomechanical properties of the ascending aorta by fitting a material model to in vivo data typically overlook this aspect [12, 27]. Using the simulated DVAS pressure–diameter relationship in Figure 1, we compared the GOH model parameter estimation when including or not the dynamic axial elongation in the ascending aorta’s kinematics. The estimated GOH model parameters were compared with the ground truth, i.e., the actual model parameters used to generate the simulated curves (Table 4). Our results suggest that neglecting the dynamic axial elongation of the vessel results in high deviations in estimated model parameters from the ground truth, thereby not enabling the capture of the true biomechanical response of the vessel. These issues can be considerably reduced if the physiological dynamic axial elongation of the ascending aorta is accounted for in the parameter estimation. Nonetheless, the accurate estimation of constitutive model parameters from limited in vivo data remains an open challenge: i.e., the number of model parameters is typically too high compared to the available mechanical information. To illustrate this, we repeated the parameter estimation while fixing an increasing number of model parameters to constant (i.e., equal for all arteries) values. The observed imperturbability of the RMSE signifies that there exist multiple combinations of four to seven model parameters that similarly reproduce the data (i.e., model parameters are not unique). Notably, a higher number of freely estimated model parameters was associated with larger deviations of parameter values from the ground truth, as well as to a larger between-subject variability. These observations suggest that relying solely on the in vivo pressure–diameter curve is insufficient for estimating the specified parameters. Utilizing accurate axial force boundary conditions would ameliorate the under-determination of the fitting process. However, acquiring the axial force acting on the ascending aorta is unattainable in vivo [21]. Furthermore, it must be noted that the limited range of the in vivo pressure–diameter curve is likely another reason for the inability to determine the parameters of the constitutive model accurately. Therefore, our results qualitatively quantify the impact of the lack of experimental data on the parameter estimation process in vivo.

### Limitations

The goal of this study was to demonstrate the importance of dynamically varying axial stretches on the biomechanical response of the ascending aorta. To provide a fundamental understanding, we assumed the ascending aorta to be an idealized single-layered, thin-walled cylindrical geometry with homogenous deformations and material properties. This approach was chosen to avoid the additional complexities that may arise from (1) working with real geometries, such as segmenting multiple geometries from medical images related to different phases of the cardiac cycle which require performing finite element analysis, and (2) accounting for the tri-layered structure of arteries, which requires independent model parametrization for the three arterial layers (intima, media and adventitia), including their residual stresses [19, 28]. While such analyses could provide further insight into the distribution of wall stresses in the ascending thoracic aorta, our simplistic model is sufficient to highlight the differences between the FAS and DVAS conditions for real aortic geometries. Moreover, it is important to acknowledge that, in this study, the chosen axial strain value (i.e., a superimposed 4% engineering strain under DVAS condition) was obtained from [17] and corresponded to intra-operative conditions. We recognize that these strain values may differ from those observed in in vivo conditions and patient-specific strains should be obtained from medical images.

In addition, to elicit the impact of dynamic axial stretching of the ascending aorta on material parameter estimation, the pressure–diameter curves utilized for fitting the parameters were generated synthetically and do not yet represent actual in vivo situations. These synthetically generated pressure–diameter curves contained an implicit and arbitrary assignment of initial in vivo fixed axial stretch (${\lambda}_{z,\text{dias}}$) value of 1.2 to all patients. It must be noted that in reality, the value of initial in vivo axial stretch may not be the same for all patients because axial stretch may vary with blood pressure (note the difference between FAS and DVAS) or due to tissue remodeling induced by age or pathology. Therefore, while performing parameter estimation, considering ${\lambda}_{z,\text{dias}}$ as a fixed parameter or an estimated parameter may affect estimation of other parameters. Finally, the assumption that the axial stretch varies linearly with pressure must be experimentally verified.

## Conclusion

The physiological axial stretching of the ascending aorta throughout the cardiac cycle has a significant impact on estimating its biomechanical behavior, as well as constitutive properties from in vivo data. Therefore, it is crucial to consider axial deformation when performing in vivo biomechanical characterization of the ascending aorta. In addition, when considering arterial health in a clinical context, it is crucial to account for volume compliance resulting from distension and axial stretching, rather than solely focusing on area compliance. This is because axial stretching significantly impacts the biomechanics of the ascending thoracic aorta. Further work is warranted to ascertain the potential effect of aging and disease on this axial deformation and the resulting impact on the biomechanics of the ascending aorta.

### Supplementary Information

Below is the link to the electronic supplementary material.

Supplementary file1 (PDF 383 kb)
