# Supplementary material for: Significance of Dynamic Axial Stretching on Estimating Biomechanical Behavior and Properties of the Human Ascending Aorta
Source: Ann Biomed Eng. 2024 Jun 5;52(9):2485–95. doi: 10.1007/s10439-024-03537-6 (PMC11329543; doi:10.1007/s10439-024-03537-6)
Supplement: Supplementary file 1 — Supplementary file1 (PDF 383 kb) [file 10439_2024_3537_MOESM1_ESM.pdf]

Supplementary Material

Significance of Dynamic Axial Stretching on Estimating Biomechanical Behavior and Properties of the Human Ascending Aorta

Shaiv Parikh[1,†], Alessandro Giudici[1,2,†], Wouter Huberts[1,3], Tammo Delhaas[1], Elham Bidar[4], Bart Spronck[1,5‡], Koen Reesink[1,‡,*]

[1] Department of Biomedical Engineering, CARIM School for Cardiovascular Diseases, Maastricht University, Maastricht, The Netherlands

[2] GROW School for Oncology and Reproduction, Maastricht University, Maastricht, The Netherlands

[3] Department of Biomedical Engineering, Cardiovascular Biomechanics, Eindhoven University of Technology, Eindhoven, The Netherlands

[4] Department of Cardiothoracic Surgery, Heart & Vascular Centre, Maastricht University Medical Centre, Maastricht, The Netherlands

[5] Macquarie Medical School, Faculty of Medicine, Health and Human Sciences, Macquarie University, Sydney, Australia

* Author to whom correspondence should be addressed

[†, ‡] These authors contributed equally to this work.

Correspondence: k.reesink@maastrichtuniversity.nl

Supplemental Tables

Results of parameter estimation while fitting model parameters by prescribing fixed axial stretch (FAS) and dynamically varying axial stretch (DVAS) conditions to the synthetic experimental data representing physiological behavior of the ascending aorta *in vivo*. Four tables are presented which display the results of parameter estimation which were obtained by progressively fitting seven, six, five and four parameters.

Table S1 – Parameter estimation results when all seven parameters are estimated.

	**DVAS condition**							
	μ [kPa]	k_1 [kPa]	k_2 [-]	α [°]	κ [-]	D_0 [mm]	$\lambda_{z,\mathrm{dias}}$ [-]	RMSE
M55	71.06	30.08	19.68	17.50	0.13	36.56	1.16	0.00007
M58	35.40	64.29	10.41	8.57	0.31	35.76	1.18	0.00004
M60	91.83	54.14	26.54	14.00	0.23	38.53	1.12	0.00005
F68	160.03	80.76	52.43	37.09	0.13	44.23	1.03	0.00003
	**FAS condition**							
	μ [kPa]	k_1 [kPa]	k_2 [-]	α [°]	κ [-]	D_0 [mm]	$\lambda_{z,\mathrm{dias}}$ [-]	RMSE
M55	149.08	64.93	110.00	16.42	0.22	37.81	1.07	0.00029
M58	180.74	81.91	73.86	0.14	0.25	43.49	1.08	0.00059
M60	198.58	49.29	203.38	23.03	0.26	40.79	1.05	0.00066
F68	199.99	0.00	264.39	27.37	0.11	42.53	1.10	0.03323

Table S2 – Parameter estimation results when six parameters are estimated.

	**DVAS condition**							
	μ [kPa]	k_1 [kPa]	k_2 [-]	α [°]	κ [-]	D_0 [mm]	$\lambda_{z,\mathrm{dias}}$ [-]	RMSE
M55	5.63	9.53	4.06	45.40	0.25	25.93	1.23	0.00042
M58	30.92	36.61	7.18	40.89	0.25	34.77	1.16	0.00002
M60	27.71	23.98	6.97	37.96	0.25	32.15	1.18	0.00003
F68	24.50	39.54	18.48	57.27	0.25	33.79	1.04	0.00005
	**FAS condition**							
	μ [kPa]	k_1 [kPa]	k_2 [-]	α [°]	κ [-]	D_0 [mm]	$\lambda_{z,\mathrm{dias}}$ [-]	RMSE
M55	133.86	95.44	132.41	5.09	0.25	37.55	1.08	0.00037
M58	180.02	82.08	73.90	0.10	0.25	43.47	1.09	0.00059
M60	199.97	47.20	211.01	28.63	0.25	40.75	1.05	0.00064
F68	116.55	0.00	207.58	10.68	0.25	38.58	1.18	0.03521

Table S3 – Parameter estimation results when five parameters are estimated.

	μ [kPa]	k_1 [kPa]	k_2 [-]	α [°]	κ [-]	D_0 [mm]	$\lambda_{z,dias}$ [-]	RMSE
**DVAS condition**								
M55	11.56	32.20	8.32	30.00	0.25	30.98	1.21	0.00046
M58	43.32	47.63	9.41	30.00	0.25	37.28	1.17	0.00002
M60	37.59	32.50	9.57	30.00	0.25	34.21	1.18	0.00002
F68	95.85	99.84	64.72	30.00	0.25	42.27	1.08	0.00005
**FAS condition**								
	μ [kPa]	k_1 [kPa]	k_2 [-]	α [°]	κ [-]	D_0 [mm]	$\lambda_{z,dias}$ [-]	RMSE
M55	123.20	100.00	156.42	30.00	0.25	36.99	1.06	0.00047
M58	179.72	95.78	98.39	30.00	0.25	43.11	1.06	0.00059
M60	174.80	41.24	172.23	30.00	0.25	40.21	1.05	0.00083
F68	99.53	0.00	182.84	30.00	0.25	37.28	1.17	0.03604

Table S4 – Parameter estimation results when four parameters are estimated.

	μ [kPa]	k_1 [kPa]	k_2 [-]	α [°]	κ [-]	D_0 [mm]	$\lambda_{z,dias}$ [-]	RMSE
**DVAS condition**								
M55	12.03	35.00	8.73	30.00	0.25	31.19	1.21	0.00047
M58	37.62	35.00	7.69	30.00	0.25	36.31	1.20	0.00009
M60	38.31	35.00	9.91	30.00	0.25	34.39	1.17	0.00003
F68	71.03	35.00	38.51	30.00	0.25	40.08	1.12	0.00010
**FAS condition**								
	μ [kPa]	k_1 [kPa]	k_2 [-]	α [°]	κ [-]	D_0 [mm]	$\lambda_{z,dias}$ [-]	RMSE
M55	68.86	35.00	68.32	30.00	0.25	34.87	1.10	0.00113
M58	92.02	35.00	34.82	30.00	0.25	39.85	1.12	0.00180
M60	161.81	35.00	151.76	30.00	0.25	39.86	1.06	0.00094
F68	0.00	35.00	200.00	30.00	0.25	44.20	1.13	0.04981
